# Antipharyngitis Effects of *Syringa oblata* L. Ethanolic Extract in Acute Pharyngitis Rat Model and Anti-Inflammatory Effect of Ir-Idoids in LPS-Induced RAW 264.7 Cells

**DOI:** 10.1155/2021/5111752

**Published:** 2021-12-10

**Authors:** Wen-Bo Zhu, Fa-Zhi Su, Yan-Ping Sun, Bing-You Yang, Qiu-Hong Wang, Hai-Xue Kuang

**Affiliations:** ^1^Key Laboratory of Chinese Materia Medica, Ministry of Education of Heilongjiang University of Chinese Medicine, Harbin 150040, China; ^2^School of Traditional Chinese Medicine, Guangdong Pharmaceutical University, 232 Outer Ring Road, University Town, Guangzhou 510006, China

## Abstract

Acute pharyngitis is an inflammation of the pharyngeal mucous membrane and submucous lymphoid tissues. Unsatisfactory treatment and repeated occurrences might cause chronic pharyngitis and other complications. *Syringa oblata* L. (*S. oblata*) is a traditional Chinese medicine that exhibited a significant therapeutic effect on various inflammatory diseases. Its commercial drug Yan Li Xiao (YLX) capsule is used as a cure for the treatment of inflammatory diseases, such as bacillary dysentery, tonsillitis, bronchitis, and acute gastroenteritis. However, studies about *S. oblata* relieving acute pharyngitis are rare. In this study, high-performance liquid chromatography (HPLC) was used to analyze the chemical profile of *S. oblata,* and the bioactive phytoconstituents were isolated and identified by nuclear magnetic resonance (NMR) and mass spectrometry. An ammonia-induced acute pharyngitis rat model was established to estimate the protective effect of *S. oblata* in vivo for the first time. The severity of pharyngitis was observed by appearance index and HE staining. The cytokines expression was performed by ELISA. Crucial proteins expression associated with TLR4/NF-*κ*B/MAPK and NLRP3 inflammasome signaling pathways were analyzed by western blotting and immunohistochemistry. Furthermore, the anti-inflammatory effect of isolated compounds was evaluated by suppressing lipopolysaccharide- (LPS-) induced NO generation and regulating the cytokines levels in RAW 264.7 cells. The results showed that *S. oblata* exhibited a protective effect in the ammonia-induced acute pharyngitis rat model, and the compounds exert potential anti-inflammatory properties against LPS-activated RAW 254.7 cells.

## 1. Introduction

Acute pharyngitis is the most common seasonal nonspecific inflammation among upper respiratory tract diseases [1–3]. Patients with pharyngitis mainly manifest severe itchy throat, cough, pain, congestion, swelling, fever, dryness, and loss of appetite [4]. Additionally, untimely treatment can lead to various complications, including tympanitis, laryngitis, nasosinusitis, bronchitis, and myocarditis [5, 6]. To date, several treatment strategies, including antibiotics, antiviral drugs, and glucocorticoid hormones, are used for pharyngitis in clinical to relieve pain. However, these drugs have potential risks and side effects [7–9]. Current research mainly focused on infectious pharyngitis induced by virus and bacteria, while both preclinical and clinical epidemiological studies for noninfectious pharyngitis caused by an allergic reaction, smoking, alcohol intake, and so on are lacking [10]. Therefore, the development of safer and more effective therapeutic alternatives for noninfectious pharyngitis was limited under this situation.

Accumulative evidence shows that pharyngitis is inflammation in the mucosa and submucosa tissue and related to immune responses [11]. Irregular immune homeostasis and repeated acute inflammatory may result in carcinogenesis of chronic ear-nose-throat disease and nasopharyngeal mucosa [12]. Toll-like receptors (TLRs), a member of the pattern recognition receptors (PRRs) recognizing pathogen-associated molecular patterns (PAMPs) in the pathogen, play a crucial role in the defense against invading pathogens [13, 14]. TLR4 is a key pattern recognition receptor in the TLRs family and activates the immune response by recognizing invasive pathogens. Myeloid differentiation primary response gene 88 (MyD88) is one of the main adaptor proteins for TLR4 promoting signal transduction. The nuclear transcription factor-kappa B (NF-*κ*B) and mitogen-activated protein kinases (MAPKs) can be activated via interaction of dimerized TLR4 with its downstream effector MyD88 to regulate the release of inflammatory cytokines for controlling inflammation, including interleukin-1*β* (IL-1*β*), interleukin-6 (IL-6), tumor necrosis factor-*α* (TNF-*α*), and other proinflammatory cytokines [15, 16]. As an important part of inherent immunity, nucleotide-binding oligomerization domain- (Nod-) like receptor family pyrin domain-containing 3 (NLRP3) plays an important role in the process of inflammatory response and disease. The activation of NLRP3 could recruit inflammasome-adaptor protein (ASC) and procaspase-1 to form a multimeric protein complex and accelerate procaspase-1 that is converted to mature caspase-1, which promote the maturation and secretion of cytokine precursors pro-IL-1*β* and further lead to inflammatory reactions [17–19]. Accordingly, the pathogenesis and progress of pharyngitis were closely related to TLR4/NF-kB/MAPK and NLRP3 inflammasome pathway [20, 21]. RAW 264.7 cells play an important role in the inflammatory processes. Once activated, inflammatory mediators, including nitric oxide (NO) and inflammatory cytokines, might be excess production to promote the inflammatory response [22].

*Syringa oblata* Lindl. has long been used to treat diseases such as vomiting, nausea, diarrhea, rheumatic pain, and kidney deficiency [23]. Modern pharmacological studies confirmed that the leaves of *S. oblata*, extensively applied to treat various inflammatory illnesses and the activities mentioned above, might attribute to iridoid glycosides, which, as physiologically active substances, showed remarkable activities [24]. Yan Li Xiao (YLX) capsule is one of the commercial medicines and mainly consists of water extract and powder of *S. oblata* leaves. It is used as a cure for the treatment of bacillary dysentery, tonsillitis, bronchitis, acute gastroenteritis, and so on. Furthermore, clinical observation demonstrated that YLX displays a higher treatment rate for children's acute infections among clinical cases [25]. However, there were no systematic studies performed to evaluate the potential effects of *S. oblata* in pharyngitis, and molecular mechanisms are still not clearly elucidated. The advantages and application of *S. oblata* as remedy in the treatment pharyngitis should be further explored.

## 2. Materials and Methods

### 2.1. Chemicals and Reagents

The HPLC grade methanol and acetonitrile were purchased from Merck (Darmstadt, Germany). Ultrapure water was deionized using a Milli-Q system (Millipore, Bedford, MA, USA). Column chromatography was carried out over Silica gel (200–300 mesh, Qingdao Marine Chemical Ltd.) and ODS (70 *μ*m, YMC Company Ltd.). Semipreparative HPLC was performed on a C18 OBD ^TM^ prep column (Waters, 250 × 10 mm, 5 *μ*m). All the other organic solvents were of analytical grade and purchased from Tianjin Lihua Chemical Reagent (Tianjin, China). YLX was obtained from Jilin Zhenghe Pharmaceutical Group Co., Ltd. (Jilin, China). Dexamethasone (DXM) was obtained from Tianjin Tianyao Pharmaceuticals Co., Ltd. (Tianjin, China). Bicinchoninic acid (BCA, P0010) protein assay kit was obtained from Beyotime (Shanghai, China). The IL-6 (E30646), IL-1*β* (E30419), interleukin-4 (IL-4, E30647), TNF-*α* (E30635), interferon-*γ* (IFN-*γ*, E30654), prostaglandin E2 (PGE2, E30446), epidermal growth factor (EGF, E30429), and cyclooxygenase-2 (COX-2, E30672) ELISA kits were obtained from Biotopped (Beijing, China). Primary antibodies including anti-*β*-actin (AC026), rabbit polyclonal anti-TLR4 (A17436), rabbit polyclonal anti-MyD88 (A16889), rabbit polyclonal anti-NF-*κ*B (A17436), rabbit polyclonal anti-NLRP3 (A5652), rabbit polyclonal anti-caspase-1 (A0964), rabbit polyclonal anti-ASC (A11433), rabbit monoclonal anti-JNK1/2/3 (A4867), rabbit phosphorylated anti-phospho-JNK1/2/3 (AP0276), rabbit monoclonal anti-ERK1/2 (A4782), rabbit phosphorylated anti-phospho-ERK1/2 (AP0472), rabbit monoclonal anti-p38 (A4771), and rabbit phosphorylated anti-phospho-p38 (AP0056) were purchased from ABclonal (Hubei, China) and used at 1 : 1000 dilution. Second antibody IRDye® 800CW goat anti-rabbit IgG (H + L) (bs-40295G-IRDye8) was purchased from Beijing Bioss Biotechnology (Beijing, China) and applied at 1 : 5000 dilution. All the other chemical reagents used in this study were of reagent grade.

### 2.2. Preparation of S. Oblata Ethanol Extract

**Figure 1:**
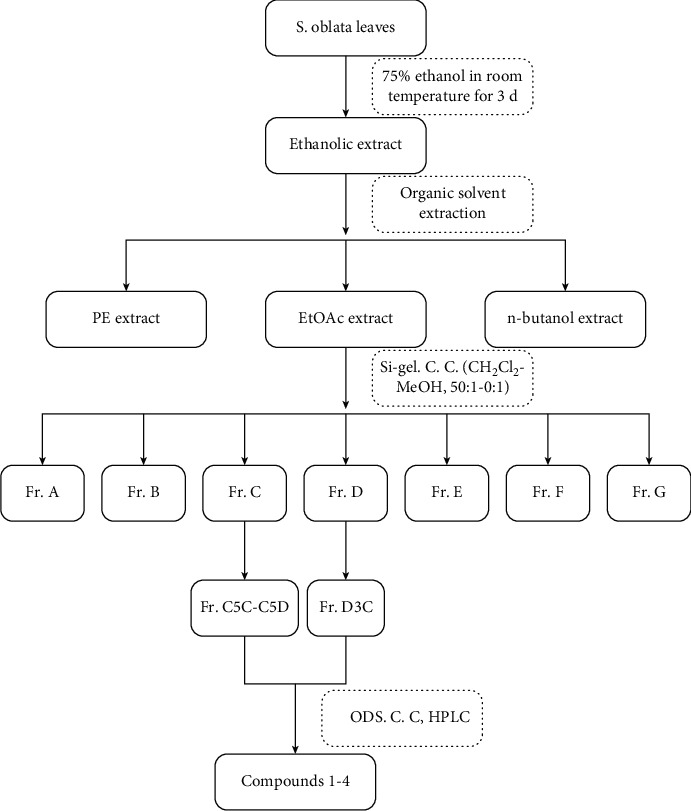
Procedure for isolation of compounds from *S. oblata* leaves.

The leaves of *S. oblata* were collected from the Heilongjiang University of Chinese Medicine. Dried leaves (408.0 g) were extracted with 75% ethanol at room temperature for 3 d by the maceration method with intermittent stirring and shaking. After filtration, ethanol was evaporated with a rotary evaporator at reduced pressure and temperature not exceeding 50°C to obtain a solid mass of extract (95.0 g). The production yield of the extract was 23.3%. Finally, the extract was dissolved in H_2_O for subsequent bioassays. The extract (80.0 g) was suspended in H_2_O (3 L) and with petroleum ether (PE) (3 × 3.0 L, 24 h each), EtOAc (5 × 3.0 L, 24 h each), and n-butanol (3 × 3.0 L, 24 h each) to give EtOAc (9.4 g) and n-butanol (3.3 g) fractions. The EtOAc-soluble portion (8.5 g) was separated by silica gel column chromatography with CH_2_Cl_2_-MeOH mixtures of increasing polarity to afford seven fractions (Fr. A-G). Fr. C-D was separated by ODS column chromatography with MeOH-H_2_O (1 : 9 to 1 : 0) to afford each five subfractions (C1–C5 and D1–D5). Subfractions C5C-C5D and D3C were purified through semipreparative HPLC on Waters SunFire® C18 OBD^TM^ Prep column (5 *μ*m, 10 mm × 250 mm) to yield compounds 1 (1.2 mg), 2 (10.4 mg), 3 (8.9 mg), and 4 (3.7 mg) (Figure 1). The structure was identified by NMR spectra, recorded on Bruker DPX 600 instrument (Bruker, Karlsruhe, Germany). HR-ESI-MS was conducted using a Waters Synapt G2-Si High-Definition Mass Spectrometer (Waters Inc., Milford, MA, USA).

### 2.3. HPLC Analysis

Liquid chromatography was performed using a Waters 2695 HPLC system equipped with a ELS detector (Waters Inc., Milford, MA, USA). The chemical profiling of *S. oblata* extract was performed using a Waters SunFire® C18 column (5 *μ*m, 4.6 mm × 250 mm). The mobile phases were A (acetonitrile) and C (waters), with gradient elution performed as 10%–80% (0–40 min). The flow rate was 1 mL/min and the injection volume was 10 *μ*L. Meanwhile, the contents of compounds isolated from *S. oblata* leaves were measured.

### 2.4. Antiacute Pharyngitis In Vivo

#### 2.4.1. Acute Pharyngitis Model Establishment and Pharmacological Intervention

**Figure 2:**
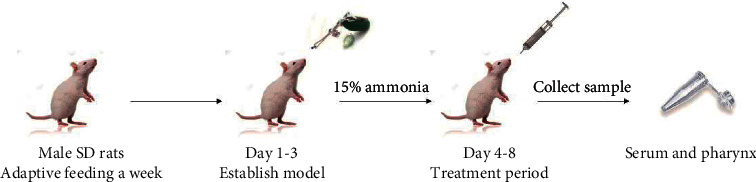
The protein expression of MyD88, p-ERK1/2, and p-p38 was detected using immunohistochemistry (×200). Control (A), model (B), DXM (C), YLX (D), *S. oblata*-H (E), *S. oblata*-M (F), and *S. oblata*-L (G). Data are expressed as mean ± SEM. ^*∗∗*^*p* < 0.01 versus control group; ^##^*p* < 0.01; ^#^*p* < 0.05 versus model group.

70 SD male rats (No. SCXK(LIAO) 2020–0001) were purchased from Liaoning Changsheng Biotechnology Co., Ltd. (Liaoning, China). The animals were feed water and food with free access for a week in a clean environment at a temperature of 25°C ± 1°C and relative humidity of 45% to 55% under 12 h light/dark cycle. All animal experiments were strictly approved by the Animal Ethics Committee of Heilongjiang University of Traditional Chinese Medicine (Heilongjiang, China) on 16 March 2021 (number 2021031604). 70 rats were randomly divided into 7 groups (*n* = 10 in each group), including the control group, model group, DEX group, YLX group, and three different doses of *S. oblata* extract groups. According to the previous study, the acute pharyngitis model was induced using a dose of 15% ammonia water sprayed into the pharyngeal mucosal twice per day for 3 consecutive days, and the rats in the control group were treated similarly with distilled water into the pharyngeal. After the model was made, the positive control group was administered with DXM (5 mg/kg). The rats in other treatment groups were administered with YLX (270 mg/kg) and *S. oblata* extracts (high: 270 mg/kg; middle: 135 mg/kg; low: 68 mg/kg), respectively. Furthermore, the rats in the control and model groups were given the same volume of distilled water. After 24 h of the last administration, all rats were killed to collect the blood and pharyngeal tissue for the following experimental indicators analysis. The experimental design is shown in Figure 2.

#### 2.4.2. Evaluation of Appearance Index

Based on behavioral and physical marking criteria [26], the changes of appearance indexes, including diet, drink, activities, mouth scratch, mouth hair loss, cough, saliva secretion, and pharyngeal swelling, were observed, and the scores were recorded using assessment criteria in Table 1.

#### 2.4.3. Cytokines Levels Assays

The blood samples were centrifuged at 3500 rpm for 10 min to obtain serum, and the pharyngeal tissues were ground with PBS (1 : 9, w/v) as a homogenization medium to obtain 10% tissue homogenate. The levels of cytokines in serum (IL-6, IL-1*β*, TNF-*α*, IFN-*γ*, IL-4) and inflammation mediators (PGE2, EGF, and COX-2) in pharyngeal were measured using enzyme-linked immunosorbent assay (ELISA) kits according to the manufacturer's instructions. The OD value was measured at 450 nm using a microplate reader, and the contents were quantified by standard curves and analyzed using GraphPad 8.0 software.

#### 2.4.4. Histological Examination of Pharyngeal

For histological evaluation, the rat pharyngeal samples were collected and fixed in 4% paraformaldehyde fixing solution for 24 h at room temperature. Then, it was embedded in paraffin and sliced. Samples were cut into 4 *μ*m thick slices and stained sequentially with hematoxylin and eosin (HE). Finally, the section was observed under a light microscope for evaluating pathological changes of pharyngeal. The severity of pathological damage was scored under blinded conditions from 0 to 4 as described in Table 2.

#### 2.4.5. Immunohistochemistry Staining of Pharyngeal

The expression of NLRP3, MyD88, p-ERK1/2, and p-p38 in pharyngeal tissue was evaluated by immunohistochemistry, and the sections used for immunohistochemistry staining came from paraffin-embedded tissues. Paraffin sections were deparaffinized into the water and repaired with antigen in the repair box of antigen repair buffer, rinsed with PBS (pH 7.4). 3% H_2_O_2_ was added to sections and incubated for 25 min at room temperature and away from light. The sections were blocked with 3% BSA for 30 min after being rinsed with PBS. The sealing fluid was gently shaken off, a portion of PBS containing the first antibodies (NLRP3, MyD88, p-ERK1/2, and P-p38) was added to the sections and incubated overnight in a humid box at 4°C, rinsed with PBS, and then incubated with a secondary antibody at room temperature for 50 min. After that, they were rewashed with PBS, and diaminobenzidine solution (DAB) was added at room temperature for 10 min. The sections were observed using a light microscope (E100, Nikon, Japan). The above process of washing with PBS was 3 times and 5 minutes each time.

#### 2.4.6. Western Blot Analysis

To elucidate the underlying mechanism of *S. oblata* in ammonia-induced acute pharyngitis model, western blotting was used to evaluate the TLR4/NF-*κ*B/MAPK and NLRP3 inflammasome signaling pathways. The total protein of pharyngeal was extracted with radioimmunoprecipitation assay (RIPA) buffer containing protease and phosphatase inhibitors. The protein concentration was measured by bicinchoninic acid (BCA) protein kit after centrifuging at 12000 rpm for 10 min. The equal amounts of protein were separated on sodium dodecyl sulfate-polyacrylamide gel electrophoresis (SDS-PAGE) and transferred to polyvinylidene difluoride (PVDF) membranes. The membrane was blocked with blocking buffer for 1 h at room temperature and then incubated overnight at 4°C with primary antibodies against TLR4 (1 : 1000), MyD88 (1 : 1000), NF-*κ*B (1 : 1000), NLRP3 (1 : 1000), caspase-1 (1 : 1000), ASC (1 : 1000), JNK1/2/3 (1 : 1000), p-JNK1/2/3 (1 : 1000), ERK1/2 (1 : 1000), p-ERK1/2 (1 : 1000), p38 (1 : 1000), p-p38 (1 : 1000), and *β*-actin (1 : 1000). The membranes were washed 3 × 5 with PBST and then incubated for 1 h at room temperature with IRDye® 800CW goat anti-rabbit IgG (H + L) second antibody (1 : 5000). After washing with PBST, the target protein bands were detected with Odyssey® CLx imaging system, and the grey density of bands was calculated by Image J software.

### 2.5. Anti-Inflammatory Activity In Vitro

#### 2.5.1. Cell Culture

RAW 264.7 cells were purchased from Stem Cell Bank, Chinese Academy of Sciences, and cultured in Dulbecco's modified Eagle's medium (DMEM) solution that contained 10% fetal bovine serum (FBS), 100 U/mL penicillin, and 100 *μ*g/mL streptomycin in a humidified atmosphere at 37°C with 5% CO_2_.

#### 2.5.2. Cell Viability

Cell viabilities were determined using the MTT method [27]. In brief, RAW 264.7 cells (1 × 10^6^ cells per well) were seeded into 96-well plates and incubated for 24 h until adhesion. Cells were treated with various concentrations of the samples (5.5, 11.0, 22.0, 44.0, 88.0, 176.0 *μ*M) for 24 h with and without treating with LPS (1.0 *μ*g/mL). After incubation, 10 *μ*L MTT solution (5 mg/mL) was added and further incubated at 37°C for 4 h. 100 *μ*L DMSO was added to each well and the absorbance was determined at 570 nm. All assays were performed in triplicate.

#### 2.5.3. No Production

The Griess method was performed to evaluate the compounds' inhibitory effects on the production of NO in RAW 264.7 cells according to the previously reported method [27]. Briefly, RAW 264.7 cells were seeded into 96-well plates, incubated for 24 h, and then induced with LPS (1.0 *μ*g/mL) in a serum-free medium containing various concentrations of the tested compounds. N-Monomethyl-larginine (L-NMMA, 5 *μ*M) was used as a positive control. After 24 h, the 100 *μ*L supernatants and NO detection kit (Beyotime, China) were mixed at 37°C. The absorbance was determined at 570 nm using a microplate reader. All assays were performed in triplicate.

#### 2.5.4. Cytokines Levels Assays

RAW 264.7 cells were plated into 96-well plates (1 × 10^6^ cells/well) and cultured for 24 h at 37°C with 5% CO_2_. After pretreating with various compounds for 24 h, the 1.0 *μ*g/mL LPS was used to stimulate RAW 264.7 cells. The supernatants were collected after 24 h and centrifuged at 12000 rpm for 10 min. The release of cytokines was measured by ELISA following the manufacturer's instructions. DXM was used as a positive control. The absorbance was determined at 450 nm using a microplate reader.

### 2.6. Statistical Analysis

Each experiment was performed at least three times, and data were presented as the mean ± standard error of the mean (SEM). The statistical significance between different groups was calculated by one-way analysis ANOVA using GraphPad 8.0 software. The value of *p* < 0.05 was considered to be significant. *p* < 0.01 was considered very significant.

## 3. Results and Discussion

### 3.1. Structure Identification

**Figure 3:**
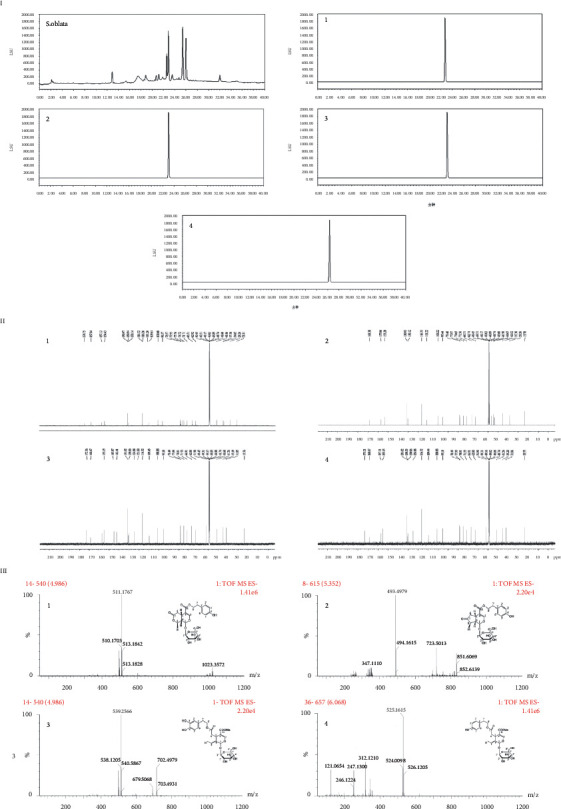
The major chemical constituents of (S) oblata. (a) Chromatographic profile of (S) oblata. by HPLC. (b) The 13C NMR spectra of four compounds (150 MHz in CD3OD). (c) The HR-ESI-MS [M–H]-of compounds group. 1: syringalactone A; 2: syringopicroside; 3: oleuropein; 4: ligstroside.

Four main compounds with sharp peaks were shown in chromatography and the retention times were 22.65, 22.99, 25.54, and 26.13 min, respectively. The chemical profile of *S. oblata* was analyzed by HPLC and is shown in Figure 3(a). ^13^C NMR spectra and HR-ESI-MS [M-H]^－^ peaks at m/z (Figures 3(b) and 3(c)) confirmed their molecular formulae. The structures of compounds were determined by comparing the NMR data with previous studies [25,28]. Additionally, the contents of four compounds were 10.21, 32.63, 28.43, and 15.83 mg/g, respectively.

Syringalactone A (1): amorphous powder; ^1^H (CD_3_OD, 600 MHz) *δ*: 7.52 (1H, s, H-3), 7.06 (2H, d, *J* = 8.5 Hz, H-2′, 6′), 6.72(2H, d, *J* = 8.5 Hz, H-3′, 5′), 5.48 (1H, d, *J* = 7.8 Hz, H-1), 4.69 (1H, d, *J* = 7.8 Hz, H-1″), 4.47 (1H, m, H-8), 4.28(2H, m, H-8′), 3.92 (1H, m, H-6″*α*), 3.64 (1H, m, H-6″*β*), 3.62 (1H, m, H-2″), 3.38 (1H, m, H-5″), 3.33 (1H, m, H-4″), 3.23 (1H, m, H-3″), 3.02 (1H, m, H-5″), 2.85 (2H, m, H-7′), 2.82 (1H, dd, *J* = 4.6, 16.5 Hz, H-6*β*), 2.44 (1H, dd, J = 11.6, 16.5 Hz, H-6*α*), 2.10 (1H, d, *J* = 7.2 Hz, H-9), 1.50 (3H, d, *J* = 7.2 Hz, H-10). ^13^C (CD_3_OD, 150 MHz) *δ*: 174.75 (C-7), 167.84 (C-11), 157.13 (C-4′), 154.43 (C-3), 130.97 (C-2′), 130.94 (C-6′), 116.32 (C-3′), 116.26 (C-5′), 109.61 (C-4), 100.68 (C-1″), 96.37 (C-1), 77.93 (C-5″), 75.76 (C-8), 74.72 (C-2″), 71.71 (C-4″), 66.51 (C-8′), 62.92 (C-6″), 41.86 (C-9), 35.30 (C-7′), 34.65 (C-6), 28.26 (C-5), 21.81 (C-10). HR-ESI-MS m/z 510.1584 [M-H]^−^ (calculated to be 510.1737).

Syringopicroside (2): amorphous powder; ^1^H (CD_3_OD, 600 MHz) *δ*: 7.44 (1H, s, H-3), 7.04 (2H, d, *J* = 8.4 Hz, H-2′, 6′), 6.72 (1H, d, *J* = 8.4 Hz, H-3′, 5′), 5.61 (1H, d, *J* = 3.2 Hz, H-1), 4.69 (1H, d, *J* = 7.9 Hz, H-1″), 4.25 (2H, m, H-8′), 3.71 (1H, m, H-6″*α*), 3.64 (1H, m, H-6″*β*), 3.39 (1H, m, H-4″), 3.22 (1H, m, H-3″), 3.21 (1H, m, H-2″), 3.20 (1H, m, H-5), 3.19 (1H, m, H-5″), 2.83 (2H, t, *J* = 6.9 Hz, H-7′), 2.54 (1H, m, H-8), 2.45 (1H, m, H-9), 2.33 (1H, m, H-6*α*), 2.11 (1H, m, H-6*β*), 1.13 (3H, d, *J* = 7.1, H-10). ^13^C (CD_3_OD, 150 MHz) *δ*: 219.81 (C-7), 168.38 (C-11), 157.06 (C-4′), 153.20 (C-3), 130.93 (C-2′, 6′), 130.12 (C-1′), 116.31(C-3′, 5′), 111.22 (C-4), 100.22 (C-1′), 95.44 (C-1), 78.40 (C-3″), 77.97 (C-5″), 74.65 (C-2″), 71.56 (C-4″), 66.31 (C-8′), 62.74 (C-6″), 46.50 (C-9), 44.65 (C-8), 35.30 (C-7′), 28.26 (C-5), 13.70 (C-10). HR-ESI-MS m/z 494.1692 [M-H]^−^ (calculated to be 494.1788).

Oleuropein (3): amorphous powder; ^1^H (CD_3_OD, 600 MHz) *δ*: 7.51 (1H, s, H-3), 5.90 (1H, br s, H-1), 4.81 (1H, d, *J* = 7.8 Hz, H-1″), 4.22 (2H, m, H-8′), 4.21 (1H, dt, J = 7.1, 10.7 Hz, H-8*β*), 4.08 (1H, dt, J = 7.1, 10.7 Hz, H-8*α*), 3.95 (1H, dd, J = 4.4, 9.2 Hz, H-5), 3.88 (1H, dd, J = 1.7, 11.9 Hz, H-6″*α*), 3.71 (3H, s, 11-OMe) 3.67 (1H, dd, J = 5.7, 11.9 Hz, H-6″*β*), 3.42 (1H, m, H-2″), 3.39 (1H, m, H-5″), 3.34 (1H, m, H-4″), 3.32 (1H, m, H-3″), 2.82 (2H, t, *J* = 7.0 Hz, H-7′), 2.71 (1H, dd, J = 4.5, 14.1 Hz, H-6*α*), 2.44 (1H, dd, J = 9.2, 14.1 Hz, H-6*β*), 1.64 (3H, d, *J* = 7.1 Hz, H-10). ^13^C (CD_3_OD, 150 MHz) *δ*: 173.36 (C-2), 168.67 (C-11), 155.15 (C-3), 145.87 (C-3′), 142.41 (C-4′), 131.02 (C-1′), 130.06 (C-9), 124.88 (C-8), 121.08 (C-6′), 116.32 (C-2′), 116.32 (C-5′), 109.43 (C-4), 100.88 (C-1″), 95.18 (C-1), 78.49 (C-5″), 77.99 (C-3″), 74.81 (C-2″), 71.53 (C-4″), 66.91 (C-8′), 62.80(C-6″), 51.90 (11-OMe), 41.26 (C-6), 35.19 (C-7′), 31.85 (C-5), 13.56 (C-10). HR-ESI-MS m/z 538.1490 [M-H]^−^ (calculated to be 538.2050).

Ligstroside (4): amorphous powder; ^1^H (CD_3_OD, 600 MHz) *δ*: 7.51 (1H, s, H-3), 7.06 (2H, d, *J* = 8.5 Hz, H-2′, 6′), 6,72 (1H, d, *J* = 8.5 Hz, H-3′, 5′), 6.07 (1H, br q, *J* = 7.0 Hz, H-8), 5.92 (1H, br s, H-1), 4.80 (1H, d, *J* = 7.8 Hz, H-1″), 4.21 (1H, dt, J = 7.1, 10.7 Hz, H-8′*α*), 4.10 (1H, dt, J = 7.1, 10.7 Hz, H-8′*β*), 3.95 (1H, dd, J = 4.5, 9.2 Hz, H-5), 3.88 (1H, dd, J = 1.7, 11.9 Hz, H-6″*α*), 3.71 (3H, s, 11-OMe), 3.67 (1H, dd, J = 5.7, 11.9 Hz, H-6″*β*) 3.41 (1H, m, H-2″), 3.40 (1H, m, H-5″), 3.33 (1H, m, H-4″), 3.31 (1H, m, H-3″), 2.83 (2H, t, *J* = 7.0 Hz, H-7′), 2.71 (1H, dd, J = 4.5, 14.1 Hz, H-6*α*), 2.44 (1H, dd, J = 9.2, 14.1 Hz, H-6*β*), 1.64 (3H, dd, J = 1.4, 7.1 Hz, H-10). ^13^C (CD_3_OD, 150 MHz) *δ*: 173.21 (C-7), 168.67 (C-11), 157.11 (C-4′), 155.15 (C-3), 131.02 (C-2′, 6′), 130.53 (C-9), 130.06 (C-1′), 124.88 (C-8), 116.32 (C-3′, 5′), 109.44 (C-4), 100.88 (C-1″), 95.18 (C-1), 78.49 (C-5″), 77.99 (C-3″), 74.81 (C-2″), 71.53 (C-4″), 66.91 (-8′), 62.80 (C-6″), 51.90 (11-OMe), 41.31 (C-6), 35.20 (C-7′), 31.86 (C-5), 13.55 (C-10). HR-ESI-MS m/z 524.1702 [M-H]^−^ (calculated to be 524.1894).

### 3.2. Antiacute Pharyngitis In Vivo

#### 3.2.1. Alleviated the Pathological Symptoms of Acute Pharyngitis Rats

**Figure 4:**
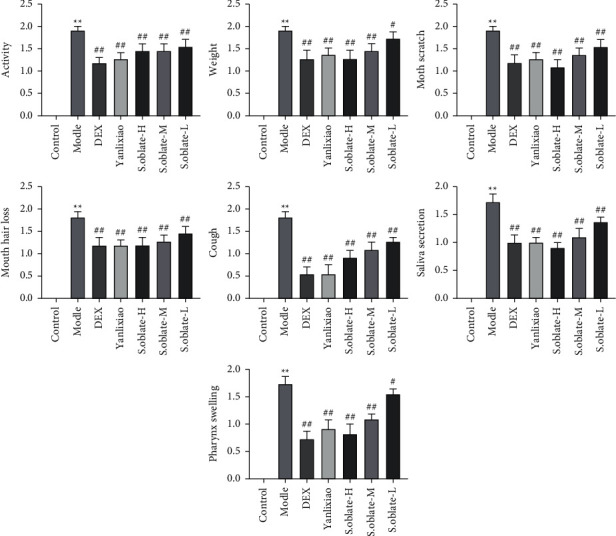
Effect of *S. oblata* on inflammatory symptoms of ammonia-induced acute pharyngitis rats. Data are expressed as mean ± SEM. ^*∗∗*^*p* < 0.01 versus control group; ^##^*p* < 0.01; ^#^*p* < 0.05 versus model group.

The severity of lesions on the pharyngeal was evaluated according to score criteria. As shown in Figure 4, rats in the control group exhibited normal activity, diet, weight, and other behaviors. However, compared with control group, the pharyngeal swelling, mucous secretions increased, the weight and activity ability of rats decreased seriously, and the mouth scratch behaviors and cough increased during modeling. Additionally, some of them lost their mouth hair gradually. As expected, these symptoms in treatment groups significantly alleviated in different degrees and reduced the pathology score compared to the model group.

#### 3.2.2. Regulating the Expression of Cytokines in Acute Pharyngitis Rats

**Figure 5:**
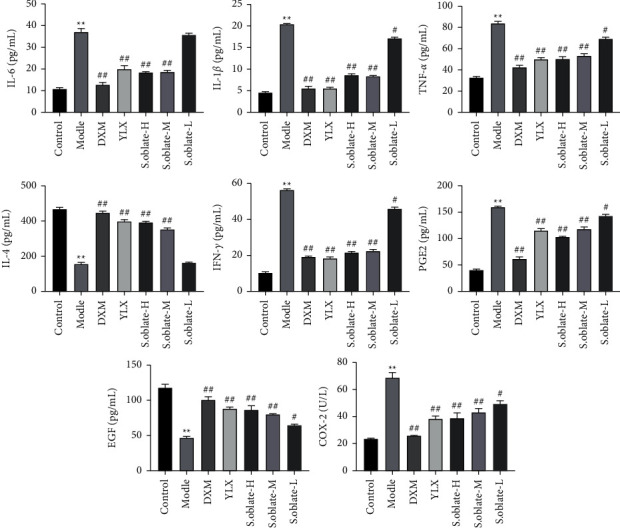
Effect of *S. oblata* on the expression of cytokines in serum and tissue. Data are expressed as mean ± SEM. ^*∗∗*^*p* < 0.01 versus control group; ^##^*p* < 0.01; ^#^*p* < 0.05 versus model group.

The expression of inflammation cytokines in serum (IL-6, IL-1*β*, TNF-*α*, IFN-*γ*, IL-4) and inflammation mediators (PGE2, EGF, and COX-2) in tissue were assessed using ELISA kits. The results showed that the levels of IL-6, IL-1*β*, TNF-*α*, IFN-*γ*, PGE2, and COX-2 were significantly increased in ammonia-induced acute pharyngitis rats compared to the control group, while the levels of IL-4 and EGF were decreased. In contrast, the balance between cytokines was regulated after treatment with *S. oblata* and YLX. The production of IL-6, IL-1*β*, TNF-*α*, IFN-*γ*, PGE2, and COX-2 was dramatically inhibited in treatment groups, whereas the release of IL-4 and EGF was promoted (Figure 5).

#### 3.2.3. Relieving Histopathological Damage in Acute Pharyngitis Rats

**Figure 6:**
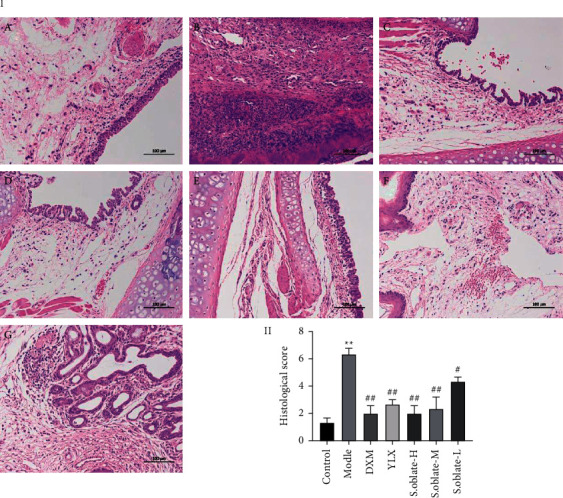
Effect of *S. oblata* on histopathological changes of ammonia-induced acute pharyngitis rats. (a) Histological evaluation of the pharyngeal (×200). (b) The histological scores were summarized. Control (A), model (B), DXM (C), YLX (D), *S. oblata*-H (E), *S. oblata*-M (F), *S. oblata*-L (G). Data are expressed as mean ± SEM. ^*∗∗*^*p* < 0.01 versus control group; ^##^*p* < 0.01; ^#^*p* < 0.05 versus model group.

Hematoxylin and eosin staining (HE) was used to evaluate the histopathological changes of pharyngeal tissue in ammonia-induced acute pharyngitis. As shown in Figure 6(a), the control group showed no evidence of damage in the pharyngeal mucosa epithelium. In contrast, an abnormal structure was observed in the model group, with massive inflammatory cell infiltration in the submucosa, connective tissue hyperplasia, and glandular atrophy. However, treated with *S. oblata* extracts, especially at a dose of 270 mg/kg, the pathological damage significantly ameliorated. Additionally, it could be observed that the microscopic scores between the control group and model group were remarkably different. The microscopic scores of *S. oblata* (270 mg/kg and 135 mg/kg) (Figure 6(b)) were lower than the model group, suggesting that the *S. oblata* alleviated acute pharyngitis in a dose-dependent manner.

#### 3.2.4. Inhibiting the Expression of TLR4/NF-*κ*B/MAPK Signaling Pathway

**Figure 7:**
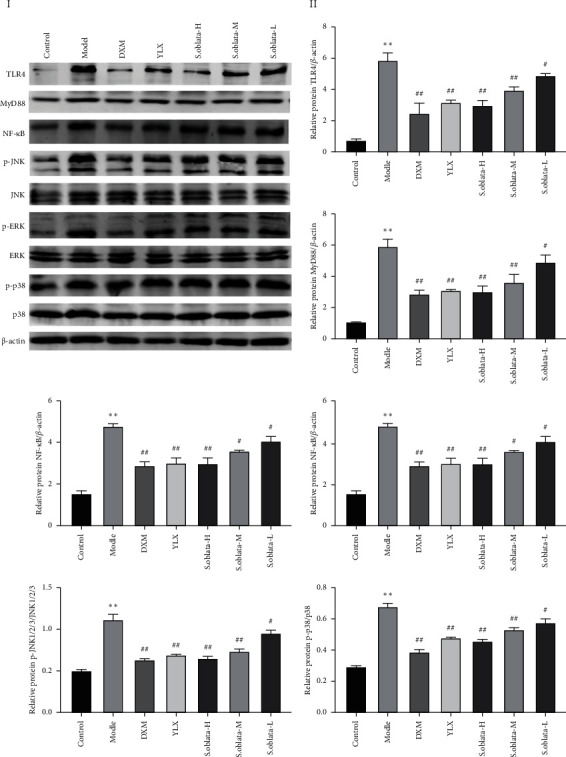
*S. oblata* suppressed the activation of the TLR4/NF-*κ*B/MAPK signaling pathway. (a) TLR4, MyD88, NF-*κ*B, JNK1/2/3, p-JNK1/2/3, ERK1/2, p-ERK1/2, p38, and p-p38 relative protein expression in acute pharyngitis rats. Data are expressed as mean ± SEM. ^*∗∗*^*p* < 0.01 versus control group; ^##^*p* < 0.01; ^#^*p* < 0.05 versus model group.

**Figure 8:**
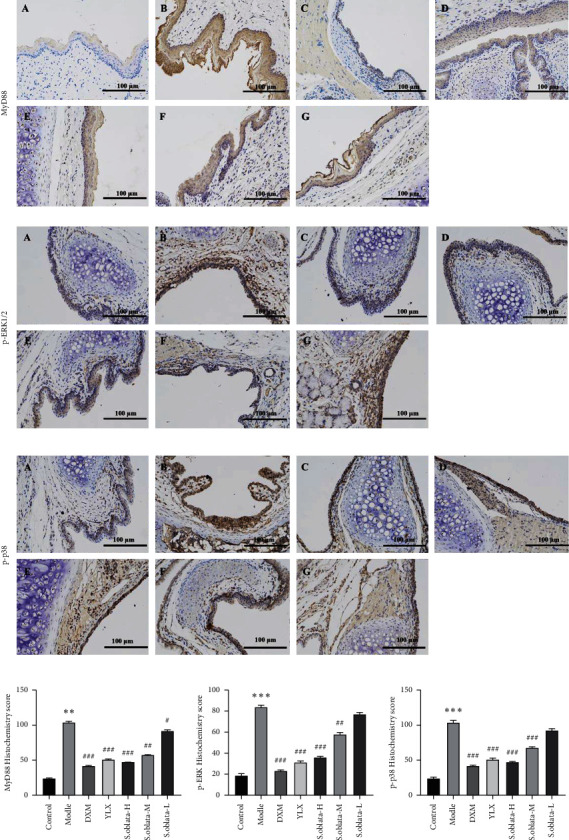
The protein expression of MyD88, p-ERK1/2, and p-p38 was detected using immunohistochemistry (×200). Control (A), model (B), DXM (C), YLX (D), *S. oblata*-H (E), *S. oblata*-M (F), and *S. oblata*-L (G). Data are expressed as mean ± SEM. ^*∗∗*^*p* < 0.01 versus control group; ^##^*p* < 0.01; ^#^*p* < 0.05 versus model group.

Inflammatory is a physiological immune response and mainly regulated via TLR4/NF-*κ*B/MAPK signal pathway in macrophages [15]. The TLR4/NF-*κ*B/MAPK signal pathway could promote and amplify the inflammatory response during cell and tissue damage and pathogen invasion. Thus, the expression of key proteins was examined by western blot and immunohistochemistry, respectively. As shown in Figures 7(a)-7(b), ammonia caused a dramatically increased in the expression of TLR4, MyD88, NF-*κ*B, JNK1/2/3, p-JNK1/2/3, ERK1/2, p-ERK1/2, p38, and p-p38 compared to the control group while the protein expression in treatment groups significantly reduced compared to the model group. Moreover, immunohistochemical staining further confirmed this result (Figure 8). The expression of MyD88, p-ERK1/2, and p-p38 was downregulated significantly by administration of *S. oblata*, suggesting that *S. oblata* alleviated acute pharyngitis via inhibiting the activation of the TLR4/NF-*κ*B/MAPK signaling pathway.

#### 3.2.5. Attenuating the Expression of NLRP3 Signaling Pathway

**Figure 9:**
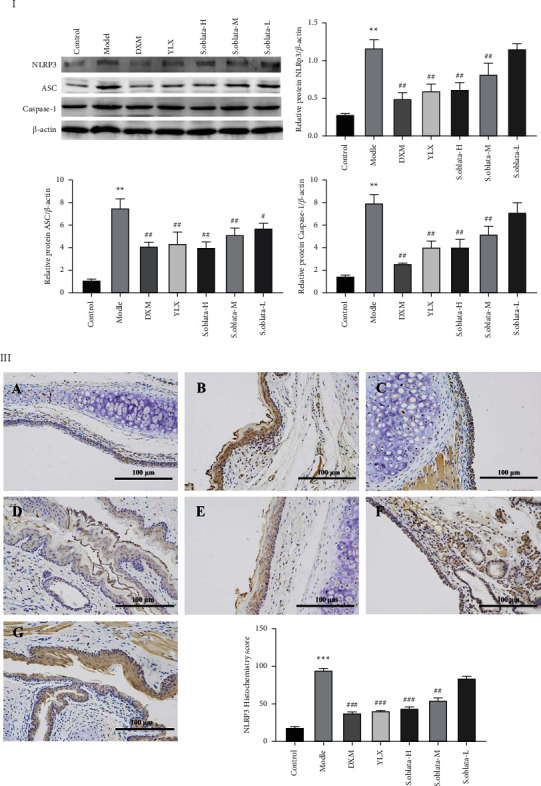
The protein expression of MyD88, p-ERK1/2, and p-p38 was detected using immunohistochemistry (×200). Control (A), model (B), DXM (C), YLX (D), *S. oblata*-H (E), *S. oblata*-M (F), *S. oblata*-L (G). Data are expressed as mean ± SEM. ^*∗∗*^*p* < 0.01 versus control group; ^##^*p* < 0.01; ^#^*p* < 0.05 versus model group.

NLRP3 inflammasome plays a critical role in the development and progression of many diseases via regulating the expression of inflammatory mediators. Thus, the expression of the related protein in the NLRP3 signaling pathway was assayed by Western blot analysis to determine the effect of *S. oblata* on NLRP3 inflammasome activity. As illustrated in Figures 9(a)-9(b), NLRP3, ASC, and caspase-1 were abnormally elevated in the model group compared with the control group, while the expression was downregulated significantly in the treatment groups. Additionally, the expression of NLRP3 was measured by immunohistochemical staining (Figure 9(c)), and a similar result was also observed, indicating that *S. oblata* reduced the acute pharyngitis by inhibiting the expression of the NLRP3 inflammasome signaling pathway.

### 3.3. Anti-Inflammatory Activity On Vitro

#### 3.3.1. Effect of Compounds on Cell Viability in RAW 264.7 Cells

**Figure 10:**
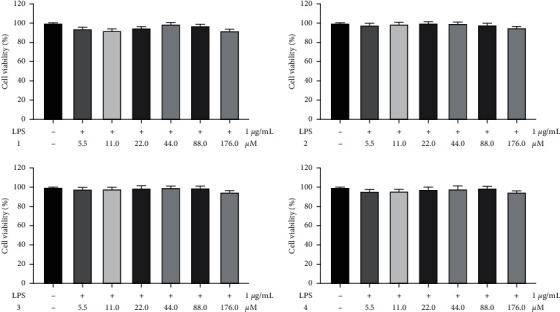
Effect of different concentrations' test compounds on cell viability in RAW 264.7 cells. Data are expressed as mean ± SEM. ^*∗∗*^*p* < 0.01 versus control group. (a) Syringalactone A. (b) Syringopicroside. (c) Oleuropein. (d) Ligstroside.

The MTT assays were used to evaluate the effect of compounds syringalactone A (1), syringopicroside (2), oleuropein (3), and ligstroside (4) on cell viability in RAW 264.7 cells. The four iridoids at the concentration ranging from 5.5 to 176.0 *μ*M had no cytotoxic in RAW 264.7 cells (Figure 10). Therefore, these concentrations of test compounds were selected for the subsequent experiments' anti-inflammatory effects.

#### 3.3.2. Effect of Compounds on NO Production in RAW 264.7 Cells

**Figure 11:**
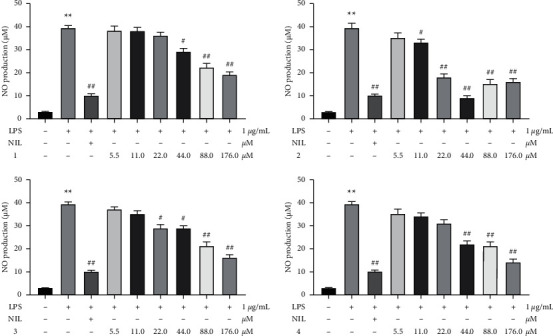
The effect of different concentrations' test compounds on LPS-induced nitric oxide (NO) production in RAW 264.7 cells. ^*∗∗*^*p* < 0.01 versus control group; ^##^*p* < 0.01; ^#^*p* < 0.05 versus model group. (a) Syringalactone A. (b) Syringopicroside. (c) Oleuropein. (d) Ligstroside.

As shown in Figure 11, test compounds significantly decreased NO production with concentrations 44.0, 88.0, 176.0 *μ*M. Furthermore, all compounds showed an evidently dose-dependent manner except for syringopicroside (2). Hence, the 44.0, 88.0, and 176.0 *μ*M were used for the analysis of other inflammatory cytokines.

#### 3.3.3. Effect of Compounds on Cytokines Levels in RAW 264.7 Cells

**Figure 12:**
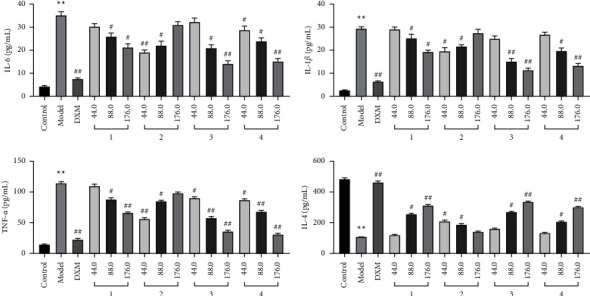
The effect of different concentrations' test compounds on LPS-induced inflammatory cytokines in RAW 264.7 cells. ^*∗∗*^*p* < 0.01 versus control group; ^##^*p* < 0.01; ^#^*p* < 0.05 versus model group.

To further investigate the inflammatory activity, the inhibitory effects of the four compounds were assessed using Elisa. The result showed that treatment of cells with LPS caused a significant elevation in IL-6 and TNF-*α* levels while decreasing the IL-4 level. However, pretreatment with test compounds (44.0, 88.0, 176.0 *μ*M) significantly reversed this situation, suggesting that the four compounds were the inhibitors of inflammation at the tested concentrations (Figure 12).

### 3.4. Discussion

*S. oblata* is a traditional herbal medicine that has anti-inflammatory property, and its commercial drug YLX capsule is extensively applied in a variety of acute and chronic inflammatory diseases [29]. The ammonia-induced acute pharyngitis rat model has been widely used as an experimental model in pharyngitis pathogenesis studies due to being highly consistent with clinical indicators as well as the characteristics of easy, reliable, and short duration. Therefore, an ammonia-induced acute pharyngitis rat model was applied to evaluate the therapeutic effects of *S. oblata* in vivo in the present study for the first time. The administration dosage was calculated by coefficient commutation of somatotypes according to the human clinical dosage of the YLX capsule. Preexperiment results indicated that the selected dosage had no obvious side effects and was suitable for further experiments. The administration of *S. oblata* could significantly ameliorate symptoms of pharyngitis in varying degrees. Additionally, HE results provide intuitive morphologically pathological characterizations that the rats treated with *S. oblata* exhibited complete epithelial structure of the mucosa and few lymphocytes infiltration.

Cytokines, mainly released by activated lymphocytes and monocytes, are acknowledged to be responsible for the initiation and progression of inflammation [30]. Particularly, IL-6, IL-1*β*, and TNF-*α* are considered the most important proinflammatory cytokines. Previous studies revealed that cytokines play a synergistic role in triggering the production of anti-inflammatory cytokines and inflammatory mediators, causing more severe inflammation [31]. Thus, the levels of cytokines were determined in serum and tissue in our study. The data showed that the levels of IL-6, IL-1*β*, TNF-*α*, IFN-*γ*, PGE_2_, and COX-2 remarkably increased, while the levels of IL-4 and EGF decreased in the model group. As predicted, the *S. oblata* displayed an anti-inflammatory effect via reversing the abnormal regulation of pro- and anti-inflammatory cytokines.

The protective mechanisms of *S. oblata* in ammonia-induced noninfectious acute pharyngitis were also explored by western blot and immunohistochemistry. TLR4/NF-kB/MAPK signaling pathways, which are responsible for the expression of inflammatory cytokines and chemokines, are the major signaling cascade in initiating and propagating inflammatory response in acute pharyngitis, as reported previously [16]. As the first TLR protein identified, TLR4 is associated with a variety of inflammatory reactions. The myD88-dependent pathway is the main signals transferred method after stimulation and the interaction between TLR4 and MyD88 in the structure domain of TIR triggers a cascade reaction of downstream signals, followed by multiple downstream signaling pathways activated such as the NF-kB and MAPK classical signaling pathways to induce the release of inflammatory cytokines [15, 16]. Three major characterized subfamilies of MAPKs, ERK1/2, p38, and JNK1/2/3 were able to activate transcription regulators of inflammation in the nucleus. The phosphorylation of p38, ERK1/2, and JNK1/2/3, related to a signaling cascade involving MAPK kinases responsible for phosphorylation and activation of the MKKK and MKK, is required for MAPK activation [32]. In this present study, the relevant protein expressions involved in TLR4/NF-*κ*B/MAPK signaling pathway, including TLR4, MyD88, NF-kB, p-JNK1/2/3, JNK1/2/3, p-ERK1/2, ERK1/2, p-p38, and p38 were upregulated in the model group compared control group, indicating that TLR4/NF-*κ*B/MAPK signaling pathway was activated by ammonia. In contrast, the levels of proteins were significantly decreased after administration for 5 days. Additionally, the effect of *S. oblata* in the dosage of 270 mg/kg and 135 mg/kg was better than that of 68 mg/kg. Thus, we speculated that *S. oblata* and Yan Li Xiao may exert a protective effect in ammonia-induced acute pharyngitis via blocking TLR-4/NF-*κ*B/MAPK signaling pathway.

NLRP3 inflammasome is an important multiprotein complex, which mainly consists of a sensor (NLRP3), an adaptor (ASC), and an effector (caspase-1). It is a major intracellular inflammatory pathway, mainly driving autoimmune and inflammatory disease processes by regulating the productions of proinflammatory cytokines [33]. Assembly of NLRP3 triggered a cascade process, including cleavage procaspase-1 into active caspase-1, and then converts the pro-IL-1*β* and pro-IL-18 into mature and biologically active IL-1*β* and IL-18 from secrete into extracellular, respectively [34]. Several studies indicated that both MAPK and NF-*κ*B play important roles in cellular responses to inflammation-induced stress and NLRP3 activation [35]. Therefore, inhibition of NLRP3 inflammasome activity has been proposed as a strategy for treating inflammatory diseases. In our study, ammonia significantly increased the protein expression of NLRP3, ASC, and caspase-1 in model control. *S. oblata* effectively downregulated the activation of NLRP3, ASC, and caspase-1, and the downstream secretion proinflammatory cytokine IL-1*β* was inhibited in dosages of 270 mg/kg and 135 mg/kg.

NO is a crucial mediator contributing to the pathogenicity of inflammation. Therefore, regulating the release of NO played an important role in the investigation of the anti-inflammatory effect of test compounds. The anti-inflammatory activity in vitro of four iridoids was determined by inhibiting NO production and regulating inflammatory cytokines levels. Interestingly, test compounds exhibited a significant effect in a dose-dependent manner except for syringopicroside. This phenomenon may be caused by the contradictory effect of syringopicroside on macrophages. The result of syringopicroside in vitro was consistent with the previous study [36]. The phenomenon might be related to the contradictory effect of syringopicroside on macrophages. Therefore, the mechanisms of compounds that display an anti-inflammatory effect need to be further investigated.

## 4. Conclusions

In this study, the ammonia-induced acute pharyngitis model was used for evaluating the therapeutic effect of *S. oblata*. *S. oblata* significantly regulated the balance between proinflammatory and anti-inflammatory cytokines. The results indicated that *S. oblata* could alleviate ammonia-induced acute pharyngitis by inhibiting the activation of TLR4/NF-*κ*B/MAPK and NLRP3 inflammasome signaling pathways. Furthermore, four iridoids were isolated from the leaves of *S. oblata* and identified by NMR methods. Results indicated that all of them exerted anti-inflammatory activity in LPS-stimulated RAW 264.7 cells in different degrees. This study provided a potential basis for future clinical treatment and research in inflammation-related diseases.

## Figures and Tables

**Table 1:** Appearance indexes score criteria in ammonia-induced acute pharyngitis rat models.

Symptom rating	Activities reduce	Weight loss	Mouth scratch	Mouth hair loss	Cough	Saliva secretion	Pharyngeal swelling
0	Normal	Normal	Normal	Normal	Normal	Normal	Normal
1	Slight	Reduce	Slight	Slight	Slight	Slight	Slight
2	Severe	Severe	Severe	Severe	Severe	Severe	Severe

**Table 2:** Pathological damage score criteria in ammonia-induced acute pharyngitis rat models.

Symptom rating	Hemorrhage	Cell infiltration	Gland atrophy	Edema	Mucosal hyperplasia
0	NO	NO	NO	NO	NO
1	Mild	Mild	Mild	Mild	Mild
2	Moderate	Moderate	Moderate	Moderate	Moderate
3	Severe	Severe	Severe	Severe	Severe
4	Very severe	Very severe	Very severe	Very severe	Very severe

## Data Availability

The data used to support the findings of this study are available on request from the corresponding author.

## Abbreviations

ASC: Adaptor protein
Caspase-1: Accelerate cysteinyl aspartate specific proteinase 1
Elisa: Enzyme-linked immunosorbent assay
HE: Hematoxylin and eosin
HPLC: High-performance liquid chromatography
IL: Interleukin
MAPKs: Mitogen-activated protein kinases
MyD88: Myeloid differentiation primary response gene 88
NF-*κ*B: Nuclear transcription factor-kappa B
NLRP3: Nod-like receptor family pyrin domain-containing 3
NMR: Nuclear magnetic resonance
PAMPs: Recognize pathogen-associated molecular patterns
PRRs: Pattern recognition receptors
TLRs: Toll-like receptors
TNF-*α*: Tumor necrosis factor-*α*.

## References

[1] Viswanatha G. L., Rafiq M., Thippeswamy A. H. M. (2014). Ameliorative effect of Koflet formulations against pyridine-induced pharyngitis in rats. *Toxicology reports*.

[2] Wen D. J., Yuan L. J., Chen T. H. (2020). Effect of Bianyanning decoction on Th1/Th2 balance of acute pharyngitis rats induced by ammonium hydroxide. *Chinese Journal of Integrated Traditional and Western Medicine*.

[3] Chiappini E., Regoli M., Bonsignori F. (2011). Analysis of different recommendations from international guidelines for the management of acute pharyngitis in adults and children. *Clinical Therapeutics*.

[4] Victoria M. V., Henry M. F., James G. C., Edwards K. M. (2011). Long-term follow-up of children with periodic fever, aphthous stomatitis, pharyngitis, and cervical adenitis syndrome. *The Journal of Pediatrics*.

[5] Ma J. L., Li X., Li C. T. (2017). Effect of Bianyan oral solution on matrix metallo proteinases-9, IL-1*β* and TNF-*α* expression in rats with acute pharyngitis. *Pharmaceutical Journal of Chinese People liberation Army*.

[6] Demeslay J., De Bonnecaze G., Vairel B. (2014). Possible role of anti-inflammatory drugs in complications of pharyngitis. A retrospective analysis of 163 cases. *European Annals of Otorhinolaryngology, Head and Neck Diseases*.

[7] Miao M., Peng M., Liu B., Bai M. (2019). Effects of compound lobelia oral liquid on acute pharyngitis rabbits model. *Saudi Journal of Biological Sciences*.

[8] Saleh N., Awada S., Awwad R. (2015). Evaluation of antibiotic prescription in the Lebanese community: a pilot study. *Infection Ecology & Epidemiology*.

[9] Melkam W., Gebremedhin H., Abrha S., Masresha B., Molla F. (2015). Glucocorticosteroids: as adjuvant therapy for bacterial infections. *International Journal of Pharma Sciences and Research*.

[10] Renner B., Mueller C. A., Shephard A. (2012). Environmental and non-infectious factors in the aetiology of pharyngitis (sore throat). *Inflammation Research*.

[11] Sun Y., Zang Z., Xu X. (2011). Experimental investigation of the immunoregulatory and anti-inflammatory effects of the traditional Chinese medicine “Li-Yan Zhi-Ke Granule” for relieving chronic pharyngitis in rats. *Molecular Biology Reports*.

[12] Xiao X., Zhang Z., Chang E. T. (2018). Medical history, medication use, and risk of nasopharyngeal carcinoma. *American Journal of Epidemiology*.

[13] He J., Han S., Li X.-X. (2019). Diethyl Blechnic exhibits anti-Inflammatory and antioxidative activity via the TLR4/MyD88 signaling pathway in LPS-stimulated RAW264.7 cells. *Molecules*.

[14] Lai J.-L., Liu Y.-H., Liu C. (2017). Indirubin inhibits LPS-induced inflammation via TLR4 abrogation mediated by the NF-kB and MAPK signaling pathways. *Inflammation*.

[15] Gao H. W., Kang N. X., Hu C. (2020). Ginsenoside Rb1 exerts anti-inflammatory effects in vitro and in vivo by modulating toll-like receptor 4 dimerization and NF-*κ*B/MAPKs signaling pathways. *Phytomedicine*.

[16] Chamanara M., Rashidian A., Mehr S. E. (2019). Melatonin ameliorates TNBS-induced colitis in rats through the melatonin receptors: involvement of TLR4/MyD88/NF-*κ*B signalling pathway. *Inflammopharmacology*.

[17] Niu X., Zang L., Li W. (2020). Anti-inflammatory effect of Yam glycoprotein on lipopolysaccharide-induced acute lung injury via the NLRP3 and NF-*κ*B/TLR4 signaling pathway. *International Immunopharmacology*.

[18] Lin Y., Yang Y. (2019). MiR-24 inhibits inflammatory responses in LPS-induced acute lung injury of neonatal rats through targeting NLRP3. *Pathology, Research & Practice*.

[19] Zhang Q., Xu N., Hu X., Zheng Y. (2020). Anti-colitic effects of Physalin B on dextran sodium sulfate-induced BALB/c mice by suppressing multiple inflammatory signaling pathways. *Journal of Ethnopharmacology*.

[20] Mu M., Hu T. Y., Li D. C. (2020). Yan-Hou-Qing formula attenuates ammonia-induced acute pharyngitis in rats via inhibition of NF-*κ*B and COX-2. *BMC Complementary Medicine and Therapies*.

[21] Zhou Z. X., Mou S. F., Chen X. Q., Gong L. L., Ge W. S. (2018). Anti-inflammatory activity of resveratrol prevents inflammation by inhibiting NF-*κ*B in animal models of acute pharyngitis. *Molecular Medicine Reports*.

[22] Ha Y. M., Chung S. W., Kim J. M. (2010). Molecular activation of NF-*κ*B, pro-inflammatory mediators, and signal pathways in *γ*-irradiated mice. *Biotechnology Letters*.

[23] Zhao M., Tang W.-X., Li J. (2016). Two new monoterpenoids from the fresh leaves of Syringa oblata. *Chemistry of Natural Compounds*.

[24] Li Y., Li Z., Li C. (2018). Evaluation of hepatoprotective activity of Syringa oblata leaves ethanol extract with the indicator of glutathione S-transferase A1. *Revista Brasileira de Farmacognosia*.

[25] Zhang S. J., Shi Z. C., Wang D., Wang J. L. (2018). Chemical constituents from leaf of Syringa oblata. *Chinese Traditional and Herbal Drugs*.

[26] Miao M. S., Chang B. J., Bai M., Bai L. (2018). Specification for preparation of animal models of acute pharyngitis (Draft). *Pharmacology and Clinics of Chinese Materia Medica*.

[27] George G., Shyni G. L., Abraham B., Nisha P., Raghu K. G. (2021). Downregulation of TLR4/MyD88/p38MAPK and JAK/STAT pathway in RAW 264.7 cells by Alpinia galanga reveals its beneficial effects in inflammation. *Journal of Ethnopharmacology*.

[28] Shen Y. C., Chen C. F., Gao J. J., Zhao C., Chen C. Y. (2000). Secoiridoids glycosides from some selected jasminum spp. *Journal of the Chinese Chemical Society*.

[29] Zhou Z. Y., Han N., Liu Z. H. (2016). The antibacterial activity of syringopicroside, its metabolites and natural analogues from Syringae Folium. *Fitoterapia*.

[30] Pasrija R., Naime M. (2021). The deregulated immune reaction and cytokines release storm (CRS) in COVID-19 disease. *International Immunopharmacology*.

[31] Akhtar M., Guo S., Guo Y.-F. (2020). Upregulated-gene expression of pro-inflammatory cytokines (TNF-*α*, IL-1*β* and IL-6) via TLRs following NF-*κ*B and MAPKs in bovine mastitis. *Acta Tropica*.

[32] Chen L., Lai Y., Dong L., Kang S., Chen X. (2017). Polysaccharides from Citrus grandis L. Osbeck suppress inflammation and relieve chronic pharyngitis. *Microbial Pathogenesis*.

[33] Swanson K. V., Deng M., Ting J. P.-Y. (2019). The NLRP3 inflammasome: molecular activation and regulation to therapeutics. *Nature Reviews Immunology*.

[34] Birrell M. A., Eltom S. (2011). The role of the NLRP3 Inflammasome in the pathogenesis of airway disease. *Pharmacology & Therapeutics*.

[35] Martinon F., Burns K., Tschopp J. (2002). The inflammasome. *Molecular Cell*.

[36] Wang Y., Zhou Z., Han M. (2020). The anti-inflammatory components from the effective fraction of Syringae Folium (ESF) and its mechanism investigation based on network pharmacology. *Bioorganic Chemistry*.

